# Supplementary material for: Analysis of 26S Proteasome Activity across Arabidopsis Tissues
Source: Plants (Basel). 2024 Jun 19;13(12):1696. doi: 10.3390/plants13121696 (PMC11207565; doi:10.3390/plants13121696)
Supplement: Supplementary file 1 [file plants-13-01696-s001.zip › plants-3028058-supplementary.pdf]

Analysis of 26S Proteasome Activity Across Arabidopsis Tissues

Jagadeesan Ganapathy§, Katherine A. Hand§, and Nitzan Shabek*

Department of Plant Biology, College of Biological Sciences
University of California Davis

Supplementary Figures

Figure S1: Extended schematic representation of the fluorogenic activity assay using two different extraction methods analyzed in this study and 26S proteasome subunit gene expression in different tissues of *Arabidopsis thaliana.*

Figure. S2: Proteasome dependent activity in each tissue of *Arabidopsis thaliana* for L1 and L2 strategies.

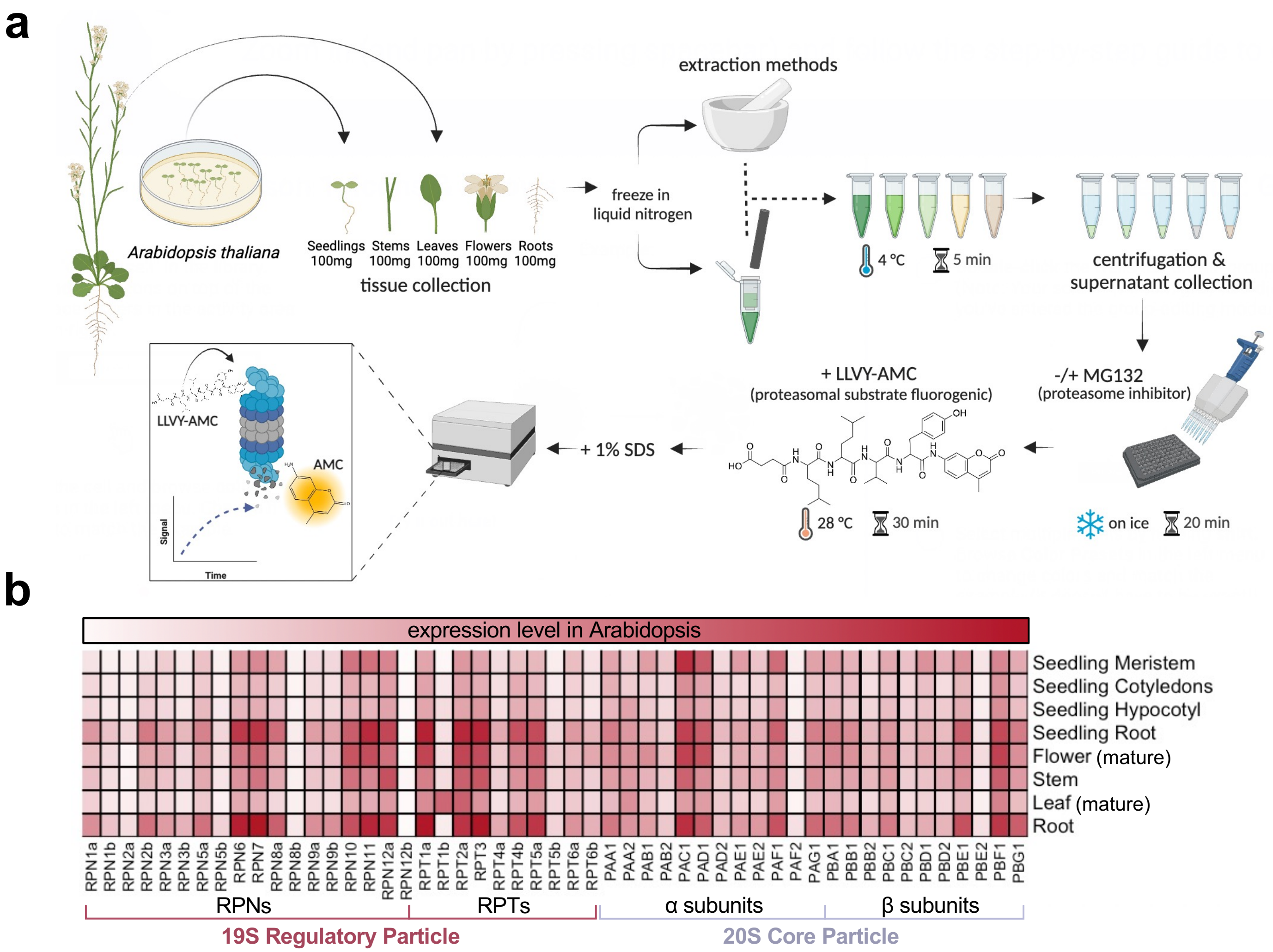

Supplementary Figure S1. (a) Extended schematic representation of the fluorogenic activity assay using two different extraction methods analyzed in this study. Image was created with BioRender.com. (b) Extended heatmap demonstrating 26S proteasome subunit gene expression in different tissues of *Arabidopsis thaliana*. Data was obtained using the Klepikova Atlas from the Bio-Analytic Resource for Plant Biology and analyzed in R-Studio. Darker red color denotes higher gene expression. TAIR accession for the 19S RP presented are: *RPN1a* (At2g20580), *RPN1b* (At4g28470), *RPN2a* (At1g08410), *RPN2b* (At2g32730), *RPN3a* (At1g20200), *RPN3b* (At1g75990), *RPN5a* (At5g09900), *RPN5b* (At5g64760), *RPN6* (At1g29150), *RPN7* (At4g24820), *RPN8a* (At5g05780), *RPN8b* (At3g11270), *RPN9a* (At5g45620), *RPN9b* (At4g19006), *RPN10* (At4g38630), *RPN11* (At5g23540), *RPN12a* (At1g64520), *RPN12b* (At5g42040), *RPT1a* (At1g53750), *RPT1b* (At1g53780), *RPT2a* (At4g29040), *RPT3* (At5g58290), *RPT4a* (At5g43010), *RPT4b* (At1g45000), *RPT5a* (At3g05530), *RPT5b* (At1g09100), *RPT6a* (At5g19990), *RPT6b* (At5g20000). TAIR accession for the 20S CP presented are: *PAA1* (At5g35590), *PAA2* (At2g05840), *PAB1* (At1g16470), *PAB2* (At1g79210), *PAC1* (At3g22110), *PAD1* (At3g51260), *PAD2* (At5g66140), *PAE1* (At1g53850), *PAE2* (At3g14290), *PAF1* (At5g42790), *PAF2* (At1g47250), *PAG1* (At2g27020), *PBA1* (At4g31300), *PBB1* (At3g27430), *PBB2* (At5g40580), *PBC1* (At1g21720), *PBC2* (At1g77440), *PBD1* (At3g22630), *PBD2* (At4g14800), *PBE1* (At1g13060), *PBE2* (At3g26340), *PBF1* (At3g60820), *PBG1* (At1g56450).

Supplementary Figure S1

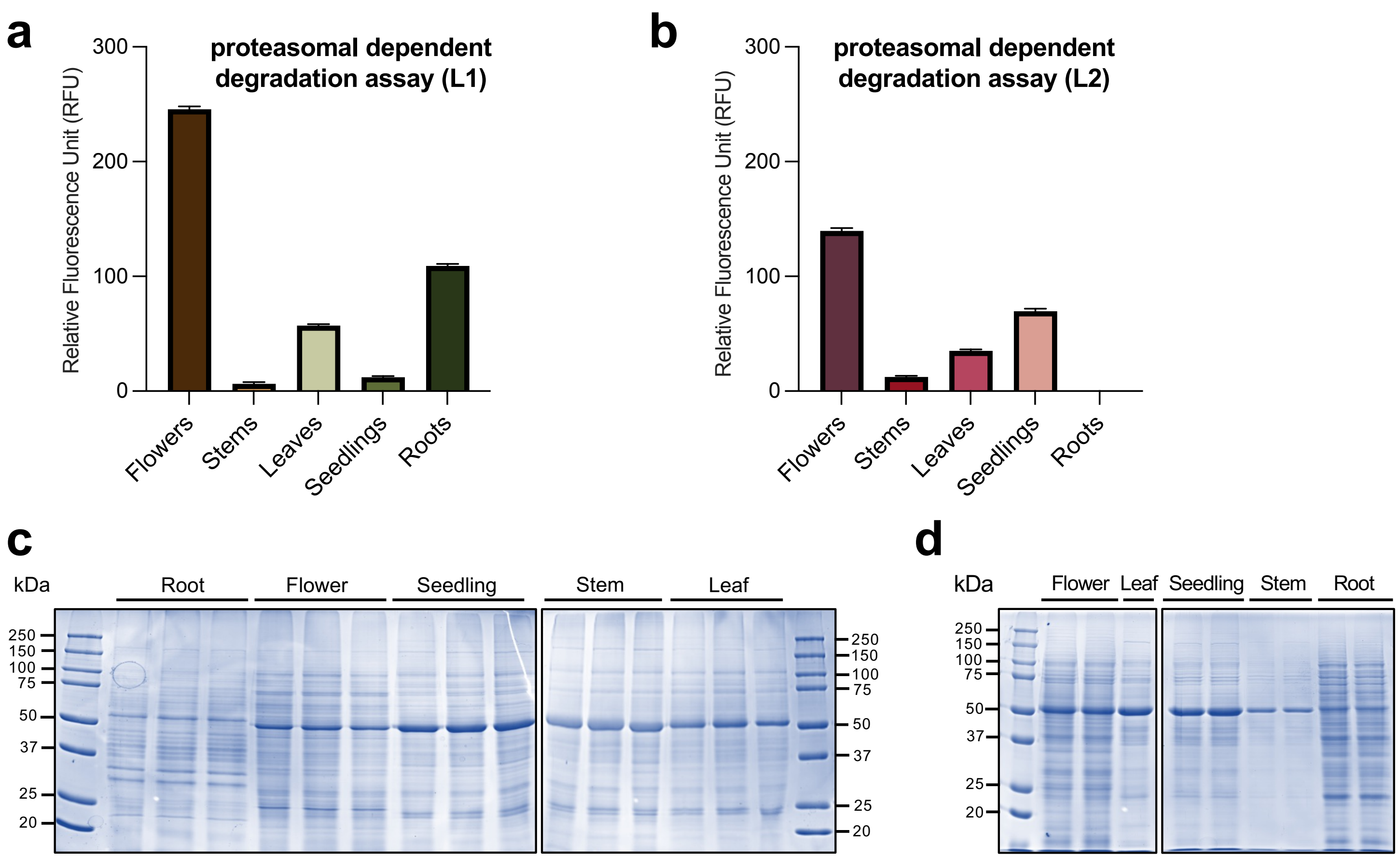

Supplementary Figure S2
